# Exacerbation of diabetic cardiac hypertrophy in OVE26 mice by angiotensin II is associated with JNK/c-Jun/miR-221-mediated autophagy inhibition

**DOI:** 10.18632/oncotarget.21302

**Published:** 2017-09-28

**Authors:** Ling-Bo Qian, Sai-Zhi Jiang, Xiao-Qiang Tang, Jian Zhang, Ya-Qin Liang, Hai-Tao Yu, Jing Chen, Zheng Xu, Rui-Ming Liu, Bradley B. Keller, Hong-Lei Ji, Lu Cai

**Affiliations:** ^1^ Cardiovascular Center, The First Hospital of Jilin University, Changchun 130021, China; ^2^ Department of Basic Medical Sciences, Hangzhou Medical College, Hangzhou 310053, China; ^3^ Pediatric Research Institute, Department of Pediatrics of the University of Louisville, Louisville, Kentucky 40202, USA; ^4^ Department of Pediatrics, The First Affiliated Hospital of Wenzhou Medical University, Wenzhou 325000, China; ^5^ Pulmonary, Allergy, and Critical Care Medicine, Department of Medicine, University of Alabama at Birmingham School of Medicine, Birmingham, Alabama 35294, USA; ^6^ Kosair Charities Pediatric Heart Research Program, Cardiovascular Innovation Institute, University of Louisville, Louisville, Kentucky 40202, USA

**Keywords:** angiotensin II, hypertrophy, diabetes, MiR-221, autophagy

## Abstract

Both diabetes and angiotensin II (Ang II) excess trigger cardiac remodeling and dysfunction, and diabetic cardiomyopathy. We hypothesized that cardiac hypertrophy associated with the development of diabetic cardiomyopathy is worsened by increased Ang II. Male type 1 diabetic OVE26 and wild-type mice were given Ang II (sc., 1.15 mg/kg, twice a day) for 14 days. Diabetes-induced cardiac dysfunction and hypertrophy was exacerbated by Ang II treatment as determined by echocardiography, wheat germ agglutinin staining and atrial natriuretic peptide. Ang II treatment dramatically exacerbated diabetes-caused decreased LC3-II, a marker of autophagy, and increased p62, an indicator of cytosolic protein clearance. Ang II treatment also augmented diabetes-associated increased phosphorylated levels of c-Jun, JNK, mTOR, and miR-221, and decreased of p27 expression, a direct target of miR-221. Chromatin immunoprecipitation assay showed that Ang II elevated c-Jun binding to the promoter of *miR-221* in diabetic mice. These results suggest that Ang II accelerates cardiac hypertrophy in the early stage of murine diabetes, probably through activation of the JKN/c-Jun/miR-221 axis and inhibition of downstream autophagy. Therefore, inhibition of Ang II or miR-221 in diabetic individuals may be a potential approach for delaying the onset and/or reducing the severity of diabetic cardiomyopathy.

## INTRODUCTION

Diabetic cardiovascular complications such as cardiomyopathy and endothelial dysfunction are the leading cause of mortality in diabetic patients. Cardiac hypertrophy often precedes the pathological phenotype of diabetic cardiomyopathy (DCM), as evidenced by the increase in heart size and mass, which ultimately leads to stiffer ventricles, irreversible cardiac remodeling, and subsequent heart failure [1]. Clinical [2] and experimental [3] results indicate that diabetes is characterized by the up-regulation of both systemic and local angiotensin II (Ang II), and interventions targeting Ang II ameliorates the pathological changes of DCM. We have previously demonstrated that short-period administration (14 days) of a subpressor dose of Ang II induces cardiac hypertrophy in both diabetic and nondiabetic mice [4], confirming that Ang II is directly involved in the development of DCM. Nonetheless, the exact mechanism by which Ang II induces the diabetic cardiac pathology remains unclear.

Currently, noncoding RNAs, such as microRNAs (miRs), have emerged as novel regulators of cardiovascular pathophysiology including cardiac hypertrophy, fibrosis, calcium handling and angiogenesis [5, 6], although the underlying mechanisms remain largely undefined. A recent report demonstrated that cardiac miR-221 is massively overexpressed in streptozotocin (STZ)-induced diabetic mice and glycemic control fails to normalize miR-221 levels [7]. Overexpression of miR-221 has been shown to cause cardiac dysfunction and heart failure accompanied with impaired autophagy through the targeted repression of p27, a cyclin-dependent kinase inhibitor, and subsequent mammalian target of rapamycin (mTOR) activation [8]. Therefore, it is believed that miR-221 may stimulate myocyte hypertrophy and block autophagy by targeting p27/mTOR signaling in the diabetic myocardium.

The effect of Ang II on cardiac expression of miR-221 *in vivo* has not been defined. Previous study has shown that miR-221 is involved in vascular smooth muscle cells proliferation [9]. In addition, miR-221 is up-regulated by Ang II in rat primary aortic vascular smooth muscle cells *in vitro* and aortas *ex vivo*, which stimulates cell proliferation [10]. We hypothesized that Ang II induces alone, and synergizes with diabetes, the up-regulation of myocardial miR-221, accelerating the development of DCM. Abundant studies demonstrated that transcription of miR-221 is under the positive control of promoter-binding transcription factor, c-Jun [11–13]. Activation of c-Jun NH_2_-terminal kinase (JNK) leads to enhanced c-Jun phosphorylation (which stabilizes c-Jun) and nuclear localization, which is essential in transcriptionally up-regulating miR-221 in cancer cell lines [11–13]. Our previous studies revealed that inhibition of diabetes-induced JNK is effective to prevent pathological changes of the heart, aorta, and kidney in STZ-induced diabetic mice [14–16]. In addition, Ang II-induced hypertrophy was recently confirmed to associate with JNK signaling activation in cardiomyocytes [17, 18]. However, whether diabetes can up-regulate miR-221 and subsequent hypertrophy via activating JNK/c-Jun signaling pathway and whether Ang II involves in this pathological process remain to be elucidated.

The present study, therefore, was designed to test our hypothesis: (a) Ang II treatment exacerbates diabetes-induced cardiac hypertrophy and remolding due to autophagy inhibition, independent of increased BP; (b) inhibited cardiac autophagy in diabetes and/or Ang II exposure may be mediated by increased miR-221 expression that further inhibits autophagy negative regulator p27 function; and (c) increased level of miR-221 in diabetes and/or Ang II condition may be mediated by diabetes- and/or Ang II-increased JNK/c-Jun-mediated transcription. Since STZ-induced type 1 diabetes (T1D) model may involve a direct STZ effect on the JNK/c-Jun/miR-221 axis, we used OVE26 mice [19], a commonly-used spontaneous T1D mouse model, in the present study. We examined the myocardial functional, pathological, and molecular changes in the diabetic OVE26 mouse model treated with and without subpressor dose of Ang II for 14 days as used in previous studies [4, 20].

## RESULTS

### General features of diabetic mice with and without Ang II treatment

**Figure 1:**
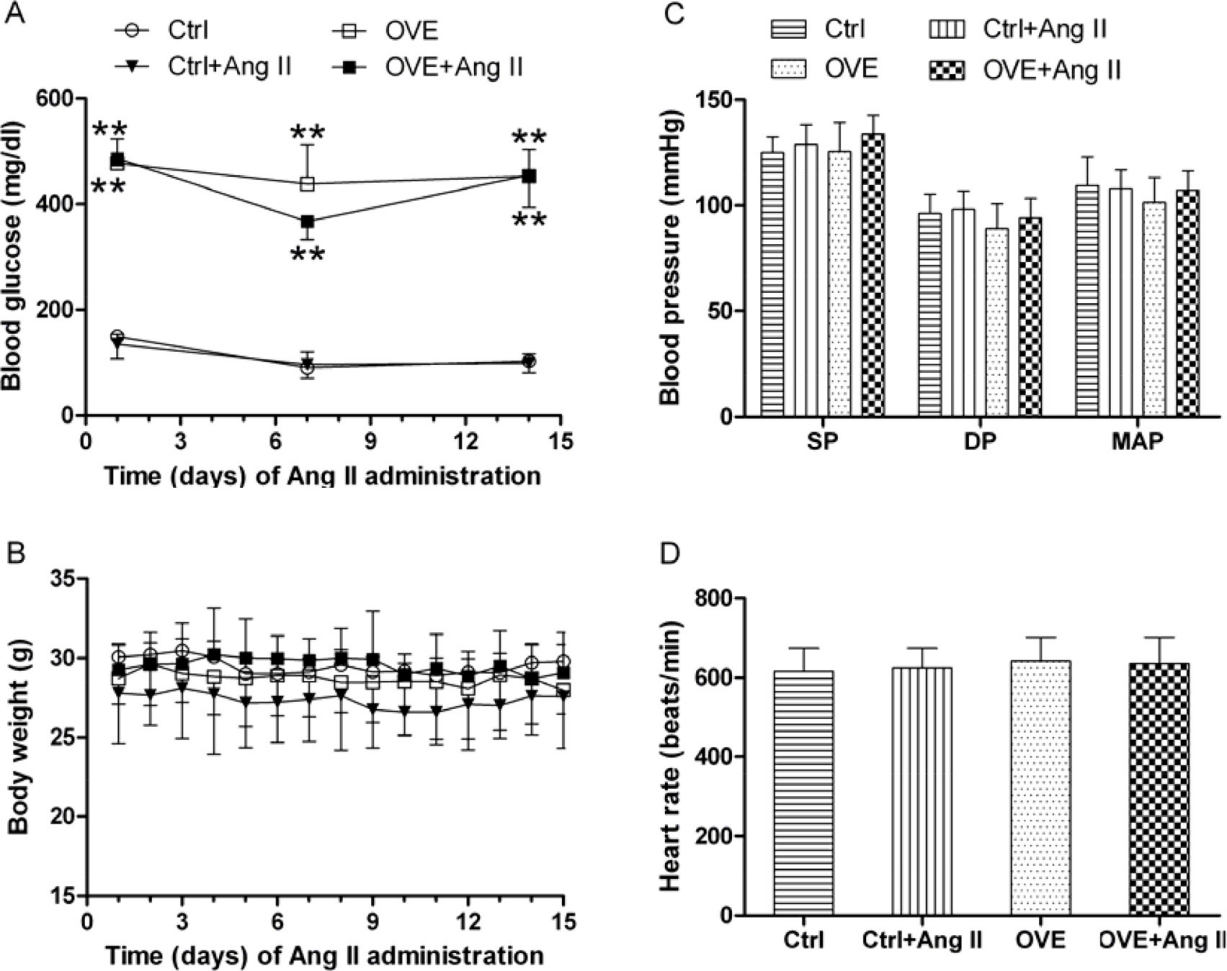
General feature of mice The dynamic blood glucose (**A**) and body weight (**B**) at different times were detected in mice. Effects of acute and subpressor Ang II (sc, 1.15 mg/kg/time, 2 times/d) on blood pressure (**C**) and heart rate (**D**) were measured in mice at the end of treatment. SP: systolic pressure, DP: diastolic pressure, MAP: mean artery pressure. Data are presented as the mean ± SD, *n* = 4 or 5 per group. ^**^P < 0.01 vs. Ctrl.

The peripheral blood glucose concentration was measured every week from tail vein whole blood and diabetes was defined by a blood glucose more than 250 mg/dl. The blood glucose values for diabetic OVE26 mice were over 350 mg/dl for the whole experiment period, indicating the presence of diabetes. Treatment with Ang II for 14 days did not significantly affect the blood glucose levels in diabetic OVE26 and or nondiabetic wild-type mice (Figure 1A), and body weight, BP, and heart rate did not change during the study period (Figure 1B–1D). These results confirmed subpressor Ang II dosing.

### Ang II exacerbates cardiac hypertrophy in diabetic OVE26 mice

Echocardiography revealed that end-systolic interventricular septal thickness (IVSs) and end-systolic left ventricular (LV) posterior wall thickness (LVPWs) significantly increased in diabetic OVE26 mice (*P* < 0.01 vs. Ctrl), similar to Ang II-treated nondiabetic mice (Table 1). Treatment with Ang II noticeably elevated diastolic IVS (IVSd), diastolic LVPW (LVPWd), IVSs, LVPWs, and LV mass in diabetic OVE26 mice hearts (*P* < 0.05 vs. Ctrl). LVPWd and IVSs further increased in Ang II-treated diabetic OVE26 mice hearts compared with the OVE26 groups (Table 1). End-diastolic LV internal diameter (LVIDd) and end-diastolic LV volume (LVVd) were moderately decreased in Ang II-treated diabetic OVE26 mice compared with non-diabetic controls (Table 1).

**Table 1:** Effect of acute Ang II on the cardiac function in diabetic OVE26 mice

	Ctrl	Ctrl+Ang II	DM	DM+Ang II
IVSd (mm)	0.64 ± 0.06	0.79 ± 0.08^*^	0.72 ± 0.07	0.80 ± 0.05^*^
LVIDd (mm)	3.45 ± 0.19	3.44 ± 0.36	3.42 ± 0.14	3.18 ± 0.01
LVPWd (mm)	0.65 ± 0.03	0.72 ± 0.06	0.66 ± 0.02	0.74 ± 0.03^*, #^
IVSs (mm)	0.79 ± 0.06	0.96 ± 0.06^**^	0.96 ± 0.07^**^	1.06 ± 0.01^**,#,+^
LVIDs (mm)	1.78 ± 0.23	1.81 ± 0.24	1.82 ± 0.03	1.83 ± 0.33
LVPWs (mm)	0.75 ± 0.07	0.92 ± 0.01^**^	0.94 ± 0.02^**^	0.96 ± 0.04^**^
LVVd (mm^3^)	45.90 ± 6.02	42.07 ± 11.85	42.66 ± 3.70	41.18 ± 6.40
LVVs (mm^3^)	9.76 ± 5.64	10.13 ± 3.35	10.13 ± 2.67	10.66 ± 0.21
EF (%)	81.79 ± 0.24	78.93 ± 1.28	81.10 ± 4.65	78.23 ± 4.36
FS (%)	46.91 ± 4.16	43.82 ± 2.87	44.54 ± 1.92	41.96 ± 3.91
LV Mass (mg)	55.44 ± 5.90	68.96 ± 4.24	68.51 ± 6.71	74.60 ± 9.79^*^

IVSd: interventricular septal thickness at diastole; LVIDd: internal dimension of left ventricular (LV) at diastole; LVPWd: LV posterior wall at diastole; IVSs: interventricular septal thickness at systole; LVIDs: internal dimension of LV at end-systole; LVPWs: LV posterior wall at systole; LVVd: LV volume at end-diastole; LVVs: LV volume at systole; EF: ejection fraction; FS: fractional shortening. Data are presented as mean ± SD, *n =* 4 or 5 per group. ^*^P < 0.05, ^**^P < 0.01 vs. Ctrl; ^#^*P* < 0.05 vs. DM; ^+^*P* < 0.05 vs. Ctrl+Ang II.

**Figure 2:**
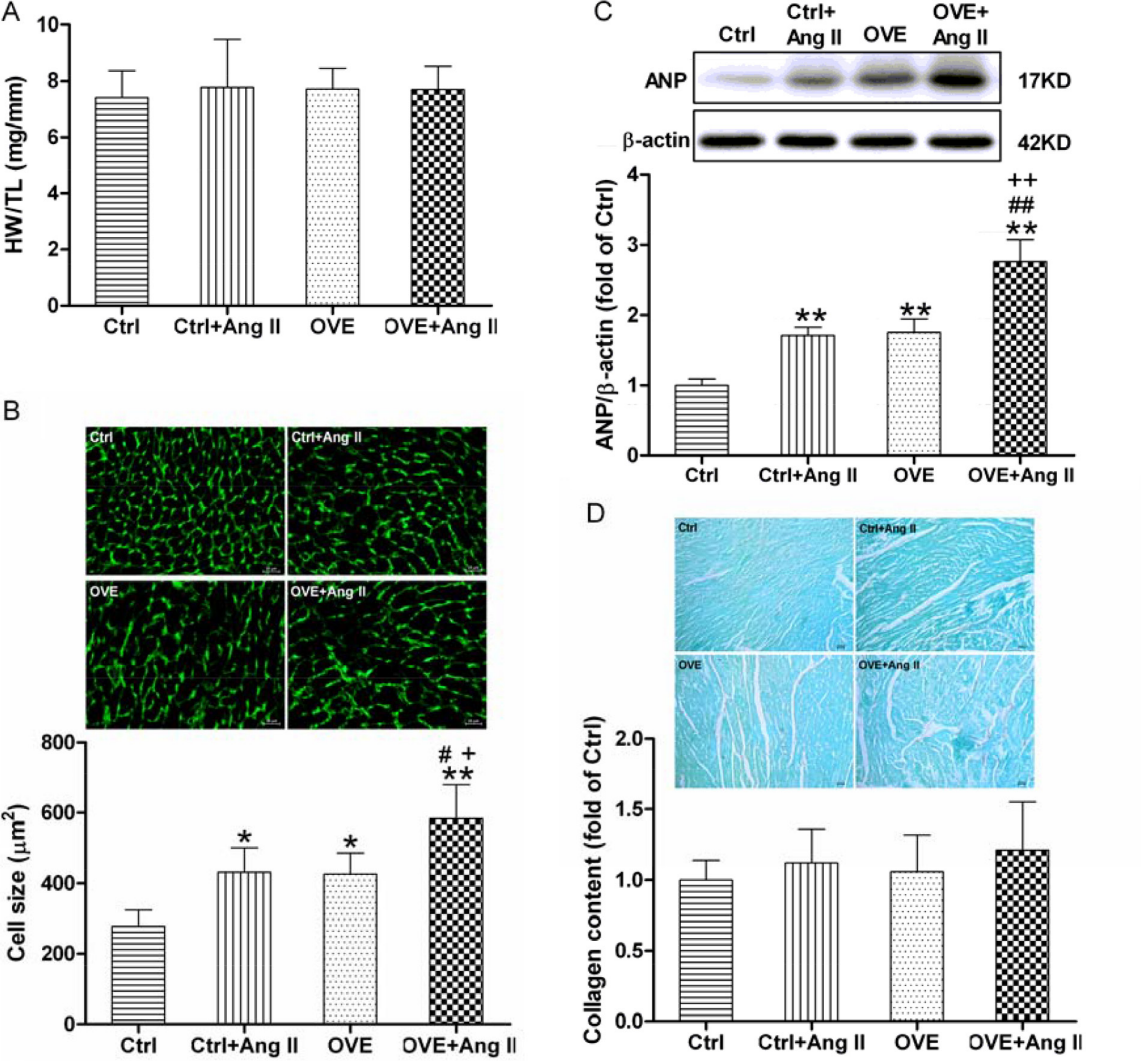
Effect of acute Ang II on the cardiac hypertrophy in diabetic OVE26 mice After 14 d of Ang II treatment, mice were euthanatized and the ratio of heart weight (HW) to tibia length (TL) (**A**) was calculated, cardiac tissue wheat germ agglutinin (WGA) staining and quantification of myocyte cross-sectional areas (**B**) were analyzed, the expression of cardiac hypertrophic marker ANP (**C**) was detected by Western blot, and Sirius red staining of collagen (**D**) was detected to estimate the cardiac fibrosis. Data are presented as the mean ± SD, *n* = 4 or 5 per group. ^*^P < 0.05, ^**^P < 0.01 vs. Ctrl; ^#^*P* < 0.05, ^##^*P* < 0.01 vs. Ctrl+Ang II; ^+^*P* < 0.05, ^++^*P* < 0.01 vs. OVE.

However, global measures of cardiac function (ejection fraction (EF) and fractional shortening (FS)) were similar between the groups (Table 1). These findings indicate a significant increase in wall thickness and a trend toward reduced internal chamber size in Ang II-treated diabetic OVE26 mice, consistent with concentric hypertrophy. The cardiac hypertrophy exacerbated by Ang II in the diabetic OVE26 mice was confirmed by noticeably enlarged cardiomyocyte size and the up-regulated cardiac hypertrophic marker atrial natriuretic peptide (ANP) (Figure 2B and 2C respectively) although there was no significant difference in heart weight to tibia length ratio or fibrosis (Figure 2A and 2D respectively).

### Ang II enhances diabetes-mediated inhibitory effect on cardiac autophagy via modulating miR-221/p27/mTOR axis

**Figure 3:**
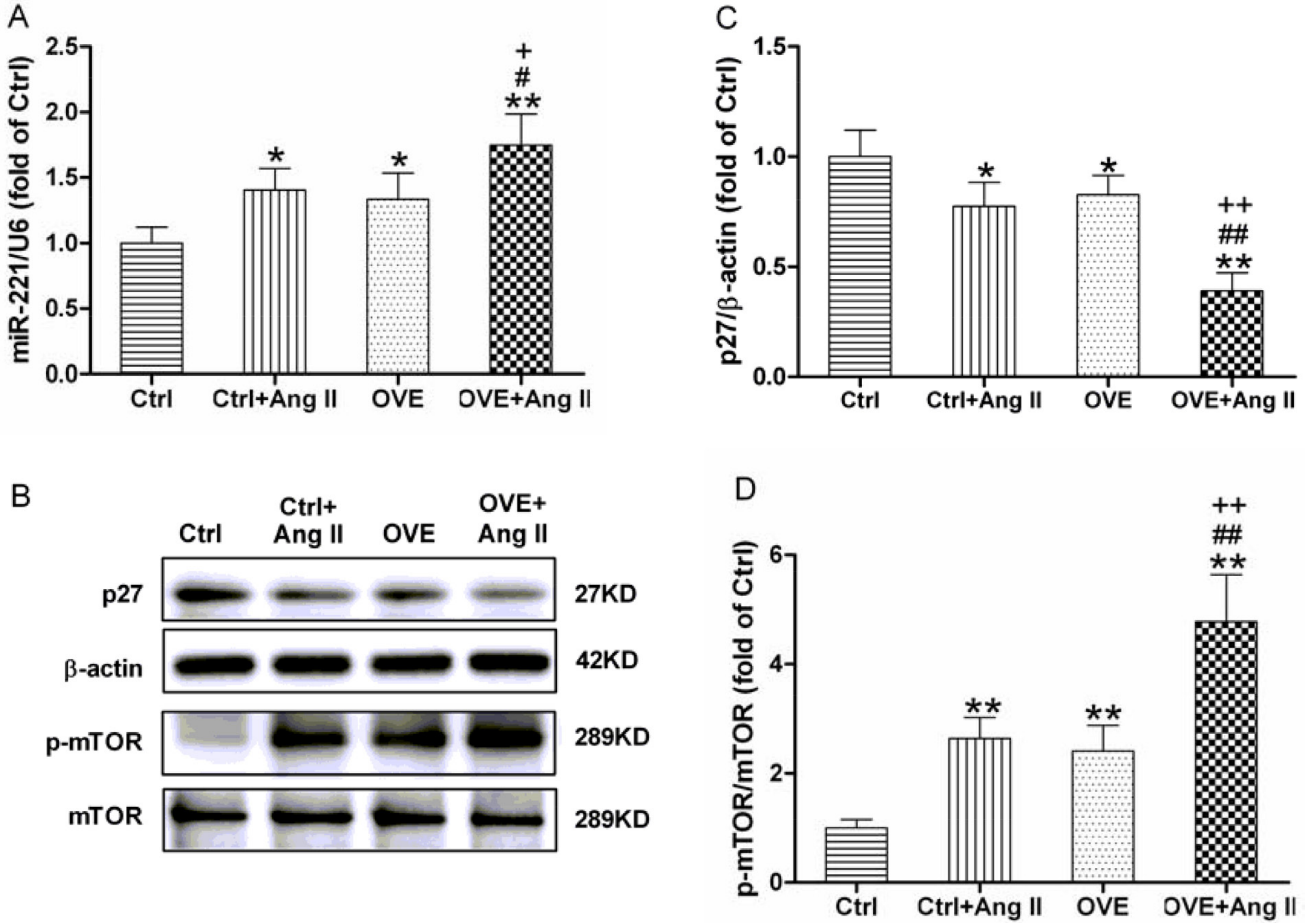
Effect of acute Ang II on the miR-221/p27/mTOR axis in diabetic OVE26 mice hearts After 14 d of Ang II treatment, miR-221 expression (**A**) was detected by quantitative PCR, p27 (**B**, **C**), the direct target of miR-221, and p-mTOR (B, **D**) were analyzed by Western blot in the mouse heart. Data are presented as the mean ± SD, *n* = 4 or 5 per group. ^*^P < 0.05, ^**^P < 0.01 vs. Ctrl; ^#^*P* < 0.05, ^##^*P* < 0.01 vs. Ctrl+Ang II; ^+^*P* < 0.05, ^++^*P* < 0.01 vs. OVE.

A significant increase in miR-221 expression was found in both the diabetic OVE26 and Ang II-treated nondiabetic mouse hearts (*P* < 0.05 vs. Ctrl), which was further enhanced in the Ang II-treated diabetic OVE26 hearts (*P* < 0.05 vs. Ctrl+Ang II, OVE, Figure 3A). As shown in Figure 3B and 3C, the expression of p27, a direct target of miR-221, was significantly repressed in the diabetic OVE26 and Ang II-treated nondiabetic mouse heart (*P* < 0.05 vs. Ctrl), and further inhibited by Ang II in the diabetic OVE26 heart (*P* < 0.01 vs. Ctrl+Ang II, OVE).

Phosphorylated mammalian target of rapamycin (p-mTOR), the downstream of p27 and a well-known negative regulator of autophagy, was significantly increased in the diabetic OVE26 and Ang II-treated nondiabetic mouse heart (*P* < 0.01 vs. Ctrl), and was further enhanced in Ang II-treated diabetic OVE26 heart (*P* < 0.01 vs. Ctrl+Ang II, OVE, Figure 3B and 3D).

**Figure 4:**
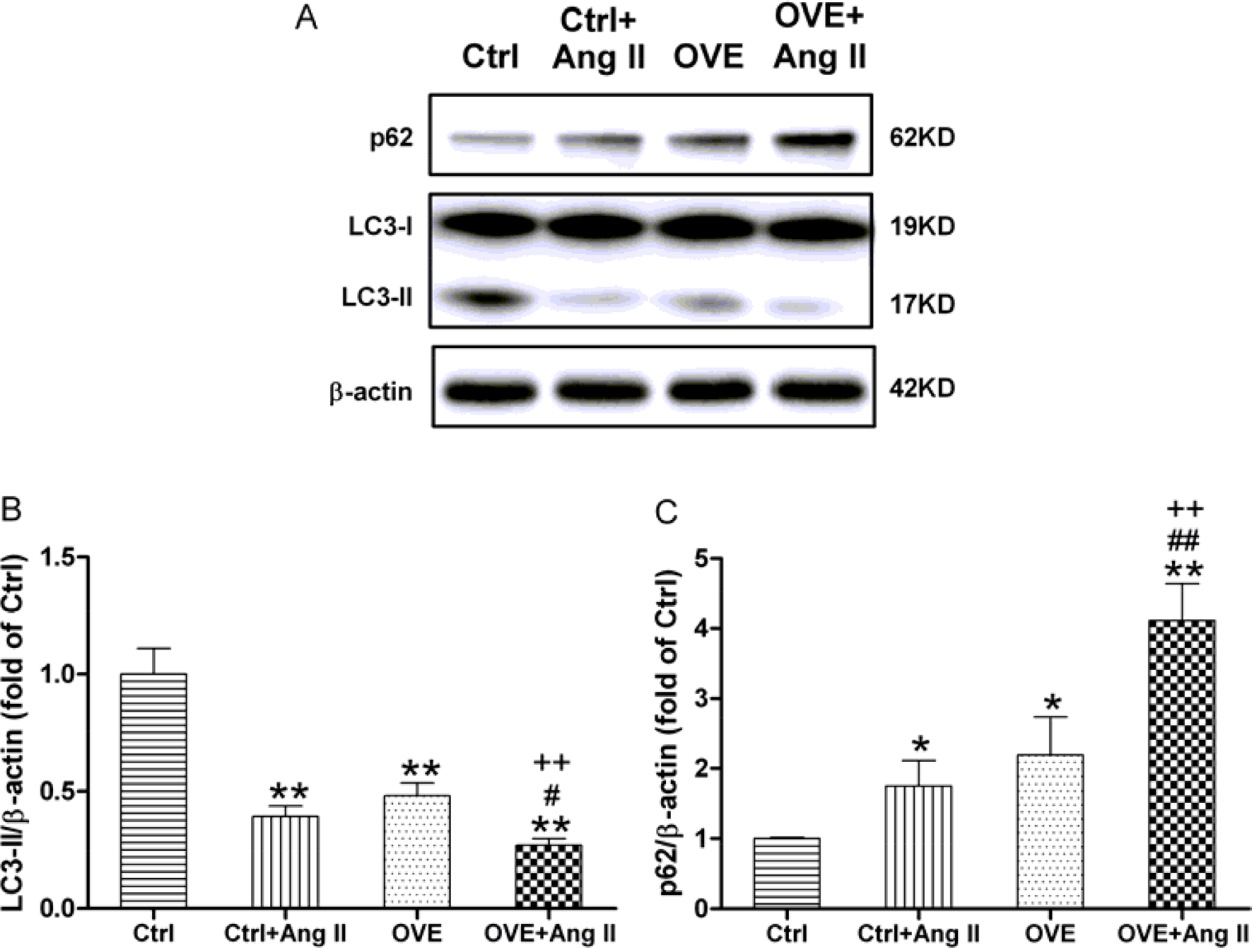
Effect of acute Ang II on the LC3 and p62 expressions in diabetic OVE26 mice hearts After 14 d of Ang II treatment, the expressions of autophagic markers LC3-II (**A**, **B**) and p62 (A, **C**) were detected by Western blot in the mouse heart. Data are presented as the mean ± SD, *n* = 4 or 5 per group. ^*^P < 0.05, ^**^P < 0.01 vs. Ctrl; ^#^*P* < 0.05, ^##^*P* < 0.01 vs. Ctrl+Ang II; ^++^*P* < 0.01 vs. OVE.

Ang II and diabetes altered two markers of autophagy, the lipidated form of microtubule-associated protein 1A/1B light chain 3 (LC3-II) and sequestosome 1 (p62). We noted decreased LC3-II and increased p62 in the diabetic OVE26 and Ang II-treated nondiabetic mouse heart (*P* < 0.01 vs. Ctrl), with greater changes in Ang II-treated diabetic OVE26 hearts (*P* < 0.05 vs. Ctrl+Ang II, OVE, Figure 4). These data suggest that Ang II and diabetes additively impair autophagy, which is associated with miR-221/p27/mTOR axis.

### Ang II and diabetes additively activate cardiac JNK/c-Jun and c-Jun binding to miR-221

**Figure 5:**
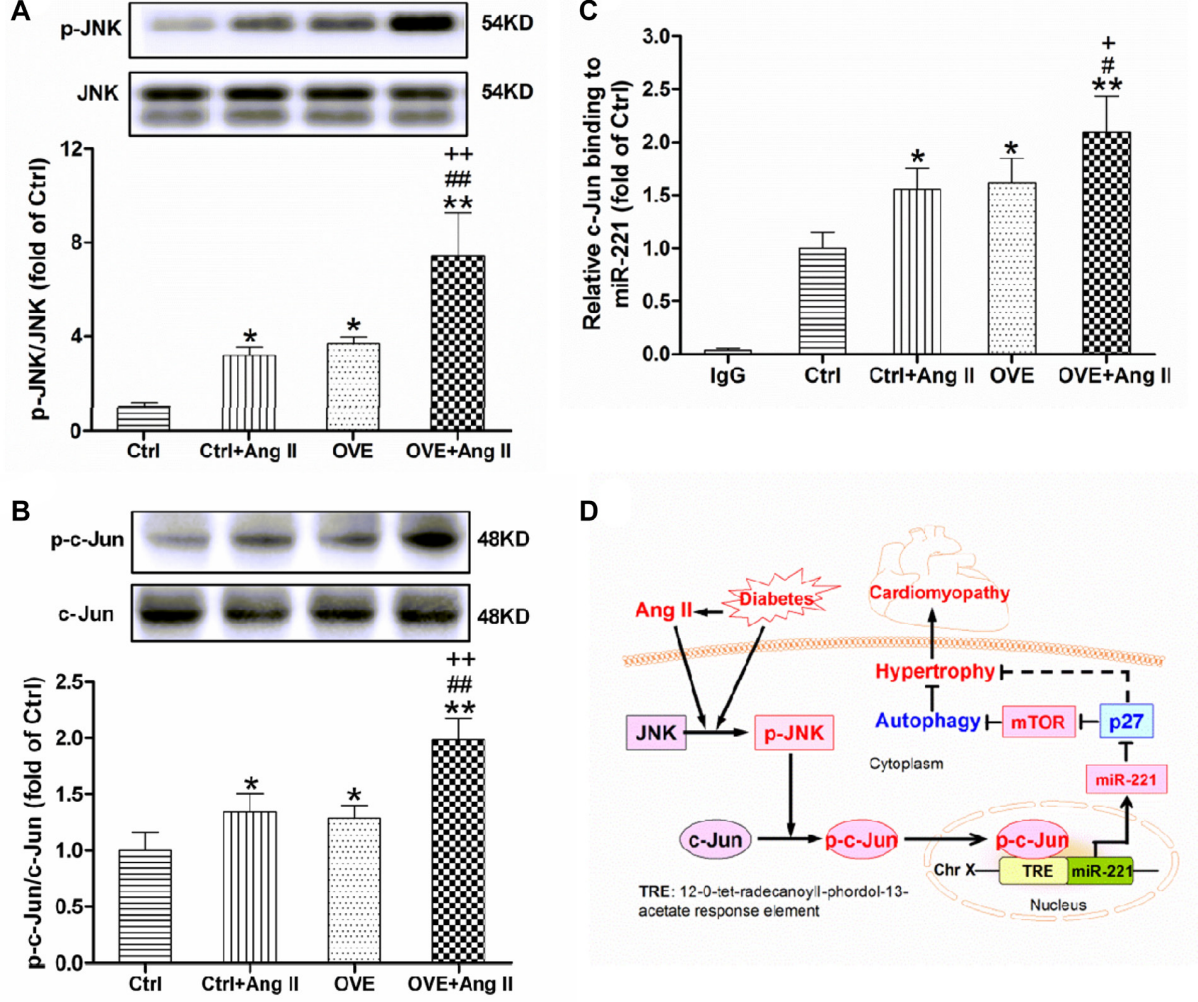
Effect of acute Ang II on the activation of JNK/c-Jun in diabetic OVE26 mice hearts After 14 d of Ang II treatment, the expressions of p-JNK (**A**) and p-c-Jun (**B**) were detected by Western blot, and c-Jun binding to miR-221 (**C**) was analyzed by ChIP in the mouse heart. Data are presented as the mean ± SD, *n* = 4 or 5 per group. ^*^P < 0.05, ^**^P < 0.01 vs. Ctrl; ^#^*P* < 0.05, ^##^*P* < 0.01 vs. Ctrl+Ang II; ^+^*P* < 0.05, ^++^*P* < 0.01 vs. OVE. Schematic illustration of the working hypothesis for the regulation of miR-221 and diabetes-induced cardiac injury by Ang II (**D**). Ang II further increases cardiac miR-221 expression through activating JNK/c-Jun and enhancing c-Jun binding to *miR-221* gene promoter, which directly inhibits p27 and then activates mTOR, leading to the inhibition of autophagy and final cardiac hypertrophy, remodeling and DCM.

A significant increase in JNK (p-JNK) expression was noted in both the diabetic OVE26 and Ang II-treated nondiabetic mouse hearts (*P* < 0.05 vs. Ctrl), and further enhanced in Ang II-treated diabetic OVE26 hearts (*P* < 0.01 vs. Ctrl+Ang II, OVE, Figure 5A). Similarly, the phosphorylation of c-Jun, a target of JKN, was noticeably enhanced in the diabetic OVE26 and Ang II-treated nondiabetic mouse heart (*P* < 0.05 vs. Ctrl), and further increased by Ang II in the diabetic OVE26 heart (*P* < 0.01 vs. Ctrl+Ang II, OVE, Figure 5B). As shown in Figure 5C, c-Jun binding was markedly enriched in the upstream of *miR-221* gene promoter in the Ang II-treated diabetic OVE26 mouse heart compared with all other groups (*P* < 0.01).

## DISCUSSION

We have demonstrated the Ang II at subpressor doses accelerates the severity of DCM observed at 6 months after diabetes onset [4]. By examining the functional, pathological, and molecular changes at this early stage (less than 3 months after the onset of diabetes) in the spontaneous T1D OVE26 mouse model [19], the present study reveals three novel findings, as illustrated in Figure 5D: (1) Treatment with acute and subpressor Ang II exacerbates diabetes-induced cardiac hypertrophy and autophagy inhibition; (2) Ang II and diabetes additively activates JNK/c-Jun-mediated up-regulation miR-221 expression; and (3) Direct down-regulation of p27 by miR-221 leads to mTOR activation and autophagy inhibition in the hearts of diabetic OVE26 and/or Ang II-treated mice, resulting in cardiac hypertrophy. These results support a pivotal role of Ang II in exacerbating pathogenesis of DCM.

Diabetes may cause heart failure by eliciting a direct detrimental impact on the myocardium leading to the development of cardiac hypertrophy, and both diastolic and systolic dysfunction. Renin-angiotensin aldosterone system blockade with angiotensin converting enzyme inhibitors or angiotensin receptor blockers has been reported to attenuate diabetes-related cardiac dysfunction without significantly affecting blood pressure [21]. However, it has been unclear whether the preventive effect of Ang II blockade on the diabetic heart was due to local myocardial or system vascular effects. Cardiac-specific overexpression of Ang II results in cardiac hypertrophy and dysfunction without hypertension in a mouse model [22], suggesting a direct effect of Ang II on the adaptive and pathologic cardiac remodeling. We have previously shown that Ang II plays a critical role in cardiac remodeling under both diabetic and nondiabetic conditions, observed at 6 months after diabetes onset [4]. In the present study, treatment of three month old OVE26 mice (development of T1D at about 2–3 weeks after birth [19] and diabetes duration of less than 3 months) with Ang II for 14 days significantly exacerbates cardiac hypertrophy.

A large body of evidence indicates that miR-mediated gene regulation is pivotal in the control of adaptive and pathological cardiac remodeling [23–25]. MiR-221 has been identified in the heart and protects cardiomyocytes against hypoxia/reoxygenation injury via theinhibition of autophagy [26]. In addition, miR-221 orchestrates the antiviral and inflammatory immune responses to viral infection of the heart [27]. Cardiac miR-221 is up-regulated in both transverse aortic constricted mice and patients with hypertrophic cardiomyopathy, resulting in increased myocyte cell size and the re-expression of fetal genes [28]. The cardiac-specific overexpression of miR-221 in mice produces cardiac enlargement and dysfunction through impairing autophagy [8] and miR-221 is up-regulated in diabetic hearts and Ang II-treated aortas [7, 10]. In the current study we demonstrated that subpressor Ang II and diabetes additively elicited cardiac hypertrophy along with increased cardiac miR-221 and inhibition of autophagosome-lysosome pathway, as evidenced by the decrease of LC3-II level and the increase of p62 level.

Autophagy plays an important role in recycling of cellular ingredients and in fuel supply during starvation; hence, it is well established that basal levels of autophagy are required for cardiomyocyte homeostasis [29]. Generally, in stress or disease conditions such as hemodynamic overload or T1D, autophagosome-lysosome pathway insufficiency occurs during, and contributes to, the maladaptive phase of cardiac hypertrophy [30, 31]. Studies have shown that inhibition of autophagy rescues DCM in type 1 diabetes [32, 33]. From detailed examination of all the available studies, an exact conclusion on the direction of cardiomyocyte autophagy modulation in response to diabetes cannot be reached [34]. Our previous study revealed that the impaired autophagy in T1D hearts associates with cardiac hypertrophy and inflammation [35], which supports the notion that appropriate levels of autophagy are required for preventing DCM. Our present data suggest that Ang II might mediate the development of DCM through the inhibition of autophagy by miR-221. Moreover, we found that p27, the direct target of miR-221, was further inhibited and that mTOR was further activated in Ang II-treated diabetic OVE26 mice. It is now recognized that p27-mediated inhibition of cyclin-dependent kinase 2 represses mTOR in cardiomyocytes [8], that p27 can function as an anti-hypertrophic factor, and that p27 is down-regulated during heart failure and cardiac hypertrophy [36]. mTOR is a pivotal upstream mediator that blocks the formation of autophagosomes by binding and inactivating the autophagy kinase complex unc-51-like kinase 1/2 [37]. Several studies have shown that mTOR is not only a pivotal inhibitor of autophagy, but also a key stimulator of dilated cardiomyopathy [37–39]. Taken together, these findings indicate that the hypertrophic effect of Ang II in diabetic hearts might be mediated, in part, by miR-221-inhibited autophagy by suppressing p27 and thereby activating mTOR [8].

It is noteworthy that Ang II activates kinase JNK through the angiotensin type 1 receptor to promote cardiomyocyte cardiac hypertrophy and apoptosis [40]. Inhibition of JNK phosphorylation also prevents high glucose-induced inflammation and apoptosis in H9c2 cell lines and primary cardiomyocytes [41]. C-Jun is usually phosphorylated by JNK and then translocates into nucleus, resulting in the transcriptional up-regulation of miR-221 in multiple cell lines [11–13]. In the present study, we found for the first time that the phosphorylation of c-Jun increased along with the activation of JNK in the Ang II-treated diabetic OVE26 mouse heart. Moreover, Chromatin immunoprecipitation (ChIP) assay confirmed that c-Jun binding to the upstream of *miR-221* gene promoter increased in the Ang II-treated diabetic mouse heart. Thus, JNK activation in the diabetic and Ang II-treated hearts is consistent with our previous study [16, 41]. These data suggest that the Ang II-induced miR-221 may be due to increasing transcriptional activity of JNK/c-Jun, and is also central for causing cardiac hypertrophy and ultimately results in DCM, as shown in Figure 5D.

There are several limitations to note in the present study. We did not repeat our investigation of the chronic effect of Ang II on diabetic hearts [4]. Additional experiments with a cardiac specific mutated c-Jun, would further provide additional evidence for our proposed role of Ang II in accentuating DCM via miR-211. The pivotal role of miR-221 in the development of DCM would also be strengthened through the use of specific inhibitors of miR-221 [8, 28]. These experiments will provide additional evidence of the role of Ang II in mediating myocardial hypertrophy and DCM.

In summary, we found that subpressor Ang II treatment exacerbated early diabetes-induced cardiac hypertrophy in OVE26 mice, which was associated with activation of JNK/c-Jun and subsequent miR-221-mediated autophagy inhibition. Our study provides insights into the development of DCM via Ang II-regulated microRNAs and implicates miR-221 as a potential therapeutic target for DCM. These findings may lead to novel therapeutic strategies for DCM.

## MATERIALS AND METHODS

### Animals

The OVE26 mouse is a well-appreciated genetically spontaneous T1D model [19]. Both male OVE26 diabetic and age- and gender-matched wild-type (WT, FVB) mice were housed at the University of Louisville Research Resources Center with a 12-h light/dark cycle at 22°C and fed on standard pellet chow and water *ad libitum*. All animal procedures were approved by the Institutional Animal Care and Use Committee of the University of Louisville, and were performed in accordance with the Guide for the Care and Use of Laboratory Animals published by the US National Institutes of Health (NIH Publication No. 85–23, revised 1996).

Twelve-week-old mice were subcutaneously injected with Ang II (1.15 mg/kg/time, 2 times/d, Sigma-Aldrich, St. Louis, MO, USA) or the same volume of vehicle (0.9% sodium chloride, 10 ml/kg/time, 2 times/d) for 14 d [20] and were divided into four experimental groups (*n* = 4–5): Age-matched nondiabetic control group (Ctrl), nondiabetic group treated with Ang II (Ctrl + Ang II), diabetic OVE26 group (OVE), diabetic group treated with Ang II (OVE + Ang II). At the end of Ang II treatment, mice were sacrificed for assaying pathological and molecular changes in the heart after performing transthoracic echocardiography and BP measurement. The dose selection was based on the following rationale. Our previous study reported that subpressor dose of Ang II infusion for 14 days stimulates cardiac hypertrophy, remodeling, and dysfunction at 5–6 months after Ang II treatment for 2 weeks, but these changes were not seen at 2 weeks after the 2-week Ang II treatment period [4]. In order to explore a potential augmented effect of Ang II on diabetes-induced cardiac hypertrophy, we selected a dose that induces hypertrophic effects seen in diabetes. Therefore we used a dose of Ang II at 1.6 μg/kg/min (equal to two injections of each 1.15 mg/kg for twice a day) for 14 days, which was shown by Nagpal et al. to induce significantly hypertrophic effects at the end of Ang II treatment for 2 weeks [20].

### Noninvasive blood pressure monitoring

BP was measured by tail cuff manometer using a CODATM noninvasive BP monitoring system (Kent Scientific, Torrington, CT, USA), based on our previous study [42]. Briefly, the BP was measured for 10 acclimation cycles followed by 20 measurement cycles. After sufficient training, formal systolic pressure, diastolic pressure and mean artery pressure were measured.

### Echocardiography

Transthoracic echocardiography was performed using a high-resolution imaging system for small animals (Vevo 770, Visual Sonics, Toronto, Ontario, Canada), equipped with a high-frequency ultrasound probe (RMV-707B). LV dimensions and wall thicknesses were measured using parasternal short axis M-mode images. EF, FS, and LV mass were calculated by using the Vevo770 software as previously published [16].

### Immunofluorescence staining

For myocyte cross-sectional area, frozen heart tissues were crosscut at 8 μm thickness in a cryostat microtome and were stained with Alexa Fluor^®^ 488 conjugated wheat germ agglutinin (WGA) (Invitrogen, Carlsbad, CA, USA). Images were captured using the Leica confocal microscope (Leica Microsystems, Heidelberg, Germany) and the cardiomyocyte size calculated using ImageJ 1.43 software as previously described [43].

### Sirius red staining

Heart paraffin sections were processed as previously described and Sirius red staining was performed to detect myocardial collagen deposition [16].

### Western blotting

Western blotting was performed according to our previous studies [16, 43]. The membranes were incubated with the primary antibodies, including ANP, β-actin (1:1000, Santa Cruz Biotechnology, Dallas, TX, USA), c-Jun, p-c-Jun, JNK, p-JNK, p62, p27 (Cell Signaling Technology, Danvers, MA, USA), mTOR, p-mTOR (1:1000, Abcam, Cambridge, MA, USA), LC3 (Novus Biologicals, Littleton, CO, USA), overnight at 4°C, washed with TBST, and incubated with the appropriate secondary antibodies for 1 h at room temperature. The protein bands were analyzed using the BIO-RAD ChemiDocTM Touch Imaging System (BIO-RAD, Hercules, CA, USA).

### Reverse transcription and quantitative PCR

As described in our previous studies [44, 45], reverse transcription and quantitative PCR for miR-221 was performed with the TaqMan MicroRNA Reverse Transcription Kit (Thermo Fisher Scientific, Grand Island, NY, USA), and the primer for miR-221 and U6 (Thermo Fisher Scientific).

### Chromatin immunoprecipitation (ChIP) assay

As described in our previous study [15], EpiQuik™ Tissue ChIP Kit (P-2012; Epigentek Group Inc., Farmingdale, NY, USA) was used to perform the ChIP assay according to the manufacturer’s protocol. In brief, the c-Jun antibody (1 μg) or 1 μl of normal mouse IgG (as a negative control) was used to pre-coat the assay wells. Meanwhile, 30 mg of heart tissue was cut into little pieces and cross-linked with 1% formaldehyde. The cross-link was stopped by glycine solution (1.25 M). After tissue disaggregation and the nuclei isolation, the DNA was sheared by sonication (S-450-Dwithmicro-tip probe; Emerson Industrial, St. Louis, MO, USA) with 5 pulses of 20 s each separated by a 40 s rest on ice (output control: 2). After centrifugation, 5 μl of the diluted supernatants were used as input DNA. The other diluted supernatant (100 μl) was added to the acetylated histone H3 antibody-coated wells followed by incubation at room temperature for 60 min. ChIP-enriched DNA fragments were precipitated, purified, and assayed by quantitative PCR with the following primers: miR-221 forward 5′-GCTAAAGAGGGGGAGCAATC-3′, reverse 5′-CTGCTCTTTGAGGGAGGACAA-3′. The value of the ChIP samples was normalized to the input and was presented as a percentage of control.

### Statistical analysis

Data are presented as the mean ± SD. Comparisons were performed by one-way ANOVA followed by the Newman-Keuls test (GraphPad Prism 5.0, San Diego, CA, USA). Statistical significance was considered as *P* < 0.05.

## Abbreviations

Ang II: angiotensin II
ANP: atrial natriuretic peptide
ChIP: chromatin immunoprecipitation
DCM: diabetic cardiomyopathy
EF: ejection fraction
FS: fractional shortening
IVSd: interventricular septal thickness at diastole
IVSs: interventricular septal thickness at systole
JNK: c-Jun N-terminal protein kinase
LC3: lipidated form of microtubule-associated protein 1 light chain 3
LV: left ventricular
LVIDd: internal dimension of LV at diastole
LVIDs: internal dimension of LV at systole
LVPWd: LV posterior wall at diastole
LVPWs: LV posterior wall at systole
LV Vold: LV volume at diastole
LV Vols: LV volume at systole
miR: microRNA
mTOR: mammalian target of rapamycin
p62: sequestosome 1
T1D: type 1 diabetes
